# Financial hardship and neighborhood socioeconomic disadvantage in long-term childhood cancer survivors

**DOI:** 10.1093/jncics/pkae033

**Published:** 2024-04-27

**Authors:** Alex J Fauer, Weiyu Qiu, I-Chan Huang, Patricia A Ganz, Jacqueline N Casillas, K Robin Yabroff, Gregory T Armstrong, Wendy Leisenring, Rebecca Howell, Carrie R Howell, Anne C Kirchhoff, Yutaka Yasui, Paul C Nathan

**Affiliations:** Family Caregiving Institute, Betty Irene Moore School of Nursing, Sacramento, CA, USA; Comprehensive Cancer Center, University of California, Davis, Sacramento, CA, USA; University of Alberta, University of Alberta, School of Public Health, Edmonton, AB, Canada; Department of Epidemiology and Cancer Control, St Jude Children’s Research Hospital, Memphis, TN, USA; David Geffen School of Medicine, University of California, Los Angeles, Los Angeles, CA, USA; Jonsson Comprehensive Cancer Center, University of California, Los Angeles, Los Angeles, CA, USA; Fielding School of Public Health, University of California, Los Angeles, Los Angeles, CA, USA; David Geffen School of Medicine, University of California, Los Angeles, Los Angeles, CA, USA; Jonsson Comprehensive Cancer Center, University of California, Los Angeles, Los Angeles, CA, USA; Surveillance and Health Equity Science Department, American Cancer Society, Atlanta, GA, USA; Department of Epidemiology and Cancer Control, St Jude Children’s Research Hospital, Memphis, TN, USA; Clinical Research Division, Fred Hutchinson Cancer Research Center, Seattle, WA, USA; Department of Radiation Physics, Division of Radiation Oncology, MD Anderson Cancer Center, The University of Texas, Houston, TX, USA; Nutrition Obesity Research Center, Department of Medicine, Division of Preventive Medicine, University of Alabama at Birmingham, Birmingham, AL, USA; Cancer Control and Population Sciences, Huntsman Cancer Institute, University of Utah, Salt Lake City, UT, USA; Department of Epidemiology and Cancer Control, St Jude Children’s Research Hospital, Memphis, TN, USA; Division of Hematology/Oncology, The Hospital for Sick Children, The University of Toronto, Toronto, ON, Canada

## Abstract

**Background:**

Long-term survivors of childhood cancer face elevated risk for financial hardship. We evaluate whether childhood cancer survivors live in areas of greater deprivation and the association with self-reported financial hardships.

**Methods:**

We performed a cross-sectional analysis of data from the Childhood Cancer Survivor Study between 1970 and 1999 and self-reported financial information from 2017 to 2019. We measured neighborhood deprivation with the Area Deprivation Index (ADI) based on current zip code. Financial hardship was measured with validated surveys that captured behavioral, material and financial sacrifice, and psychological hardship. Bivariate analyses described neighborhood differences between survivors and siblings. Generalized linear models estimated effect sizes between ADI and financial hardship adjusting for clinical factors and personal socioeconomic status.

**Results:**

Analysis was restricted to 3475 long-term childhood cancer survivors and 923 sibling controls. Median ages at time of evaluation was 39 years (interquartile range [IQR] = 33-46 years and 47 years (IQR = 39-59 years), respectively. Survivors resided in areas with greater deprivation (ADI ≥ 50: 38.7% survivors vs 31.8% siblings; *P* < .001). One quintile increases in deprivation were associated with small increases in behavioral (second quintile, *P* = .017) and psychological financial hardship (second quintile, *P* = .009; third quintile, *P* = .014). Lower psychological financial hardship was associated with individual factors including greater household income (≥$60 000 income, *P* < .001) and being single (*P* = .048).

**Conclusions:**

Childhood cancer survivors were more likely to live in areas with socioeconomic deprivation. Neighborhood-level disadvantage and personal socioeconomic circumstances should be evaluated when trying to assist childhood cancer survivors with financial hardships.

Despite advances in treatment leading to high rates of long-term survival, a childhood cancer diagnosis can lead to lasting clinical and socioeconomic challenges for survivors (1,2). The physical and psychological consequences following cancer diagnosis and treatment in childhood have been well described (3). An expanding body of literature has demonstrated the impact of personal socioeconomic status (SES) challenges, including financial hardship, on health outcomes such as quality of life and mortality (4-10). Adult survivors of childhood cancer report significantly more financial worry and food insecurity than age-matched adults without a cancer history (4). Late treatment effects are associated with disruptions in education and employment, which increase the risk of financial hardship later in life (11,12). As it is estimated that there are greater than 0.5 million childhood cancer survivors in the United States (13,14), understanding the financial burden experienced by this population is necessary.

Insufficient evidence exists to identify financial hardship risk factors in childhood cancer survivors beyond individual SES, such as health insurance coverage, educational attainment, and income (15). Research seldom focuses on the potential impact of neighborhood-level socioeconomic disadvantage on financial hardship, despite the growing literature on the association of neighborhood-level area deprivation with survival outcomes (16,17). Residing in disadvantaged neighborhoods has been linked to financial hardship in adult cancer survivors (18). Proxy measures of socioeconomic disadvantage such as the Area Deprivation Index (ADI) aggregate many social risk factors into one metric (19). Residents of disadvantaged neighborhoods may face clinician scarcity, leading to a decreased likelihood of having a regular source of health care (ie, delayed care) and incurring higher transportation expenses when seeking care (20). To add to this emerging literature, we examined the association between childhood cancer survivors’ perceived financial hardship and neighborhood-level measures of socioeconomic disadvantage.

Leveraging long-term follow-up data from the Childhood Cancer Survivor Study (CCSS), our study tested the hypothesis that neighborhood socioeconomic disadvantage is associated with self-reported financial hardship among long-term survivors of childhood cancer in comparison with a control group of siblings.

## Methods

### Study design and setting

This study analyzed cross-sectional data collected by the CCSS (15,21). Initiated in 1994, the CCSS is a 31-institution, retrospectively established North American cohort study with longitudinal follow-up aimed at determining the health outcomes of adult survivors of childhood cancer. Eligible survivors (approximately 25 000) had a confirmed diagnosis of cancer between 1970 and 1999, were aged younger than 21 years at diagnosis, and had survived at least 5 years from diagnosis (22,23). CCSS includes siblings as a comparison group. The CCSS was approved by the institutional review boards at all participating sites, and participants provided written informed consent. The UC Davis institutional review board administration reviewed this current analysis of data and determined it did not require full review (institutional review board #2068405).

CCSS collected data on the personal sociodemographics (eg, age at survey, sex, self-reported race and ethnicity, health insurance coverage, employment status, educational attainment, incomes), lifestyle (eg, physical activity, smoking status), psychological distress, and chronic health conditions from its baseline and follow-up questionnaires. Treatment data were abstracted from medical records. Participants’ home addresses were available to the researchers at the same time as the financial hardship data were linked to neighborhood adversity data.

### Participants

Data were obtained from CCSS participants in the United States who completed a follow-up survey between 2017 and 2019. Survey questions assessing financial hardship were administered to a randomly selected subset (approximately 33%) of eligible CCSS survivors (n = 3349) and siblings (n = 976). This analysis was restricted to participants who were aged 26 years or older, an age at which they can no longer be covered by parental health insurance policies under the Affordable Care Act.

### Outcome measures

The primary outcome was financial hardship, measured using 20 binary (yes, no) and Likert scale (always, usually, sometimes, rarely, never) survey items over 3 domains: behavioral hardship (coping behaviors to manage medical expenses), material hardship and financial sacrifice (conditions that arise from medical expenses), and psychological hardship (worries about medical expenses and insurance); see Supplementary Table 1 (available online) (3,4). The questionnaire items were derived from multiple national surveys (ie, National Health Interview Survey, Behavioral Risk Factor Surveillance System) for which the content validity had been cognitively tested with young adult survivors of childhood cancer, and the structural validity had been established (4). The scores of each participant’s financial hardship domain were scaled using a weighted method that accounts for the strengths of individual items with the corresponding hardship domain. The survivor and sibling scores were standardized by the survivor and sibling standard deviations, respectively.

### Neighborhood-level socioeconomic disadvantage measures

We used the ADI and Distressed Communities Index (DCI) to measure neighborhood-level adversity. The ADI is a census block–based measure, and the DCI is a county-based measure. The ADI used 17 items from the US Census to capture neighborhood-level socioeconomic disadvantage related to education, income and employment, housing, and household characteristics, which we linked to our data by 12-digit Federal Information Processing System codes (19). The ADI provides a national standardized continuous summary score from 0 percentile (least disadvantage) to 100 percentile (high disadvantage). The 2019 ADI measure was linked to the CCSS data using a crosswalk file with Federal Information Processing System codes and 9-digit zip code. The DCI is a 7-item composite index used to classify geographic variations in economic prosperity (ie, economic distress) (24). The DCI provides scores for counties based on economic activity indicators, including percent of county residents without a high school diploma, poverty rate, adults not working, housing vacancy rate, median household income, change in employment, and change in establishments. The score of each indicator reflects the percentile rank, and all indicator scores were summated and normalized to a final score ranging from 0 (most prosperous) to 100 (most distressed). A DCI score of 80 or greater represents a distressed community. The 2018 DCI data were used in this study.

### Statistical analyses

Demographic and clinical characteristics were described using frequencies, percentages, medians, interquartile ranges (IQRs), means, and standard deviation as appropriate. Demographic, clinical, and neighborhood characteristics were compared between survivors and sibling controls with Wilcoxon test for continuous variables (testing for medians) or χ^2^ test for categorical variables. Associations of area-level socioeconomic disadvantage and financial hardship among survivors were assessed with multiple linear regression models, adjusted for sex, race and ethnicity, personal income, marital status, educational attainment, employment status, health insurance, cancer diagnosis, and treatment type (anthracycline, alkylating agent, and radiation). We created quintile cut points with 20% of the population’s ADI and DCI scores for our analyses rather than examining the continuous scores. Regression models accounted for undersampling of acute lymphoblastic leukemia in the expansion cohort (1987-1999) of the CCSS; analyses were also adjusted for cubic splines (5 knots at 30, 35, 40, 50, and 55 years) of age at the time of questionnaire. All tests were 2-sided, and the alpha level of .05 was used. Analyses were conducted using SAS (version 9.4, SAS Institute Inc, Cary, NC, USA), and visualizations used R statistical package (R Core Team [2022]).

## Results

### Cohort characteristics

The analysis included 3475 survivors and 923 sibling controls who met the eligibility criteria and had nonmissing data. Characteristics of the participants are shown in Table 1. The median age at cancer diagnosis was 8 years (IQR = 4-13 years) in survivors. The median age at follow-up in survivors was 39 years (IQR = 33-47 years) in survivors and 46 years (IQR = 39-54 years) in siblings. A statistically significant proportion of survivors (37.8%) reported greater annual household incomes less than $60 000 compared with sibling controls (22.1%; *P *<* *.001). More survivors than siblings reported no health insurance coverage (8.5% vs 4.9%; *P *<* *.001). Compared with nonrespondents, survivors who responded were significantly more likely (*P* < .05) to be female, non-Hispanic White, college graduates, and married, but did not differ in terms of cancer diagnosis or receipt of alkylating or anthracycline agents as previously reported (4).

**Table 1.** Descriptive statistics of survivor and sibling individual factors^a^

Characteristic	Survivors	Siblings	*P* ^b^
	(n = 3475)	(n = 923)	
Age at survey, median (IQR), y	39.1 (33.4-46.6)	46.6 (39.1-53.9)	<.001
Sex, No. (%)			
Males	1682 (48.3)	387 (41.9)	<.001
Females	1793 (51.7)	536 (58.1)	
Race and ethnicity,^c^ No. (%)			
Black, non-Hispanic	149 (4.3)	15 (1.7)	<.001
Hispanic	220 (6.8)	29 (3.3)	
Missing	14	37	
Non-Hispanic unknown race	107 (3.5)	22 (2.5)	
White, non-Hispanic	2985 (85.4)	820 (92.6)	
US region, No. (%)			
Northeast	702 (19.8)	202 (21.9)	.095
South	1128 (33.0)	267 (28.9)	
West	768 (22.0)	218 (23.6)	
Midwest	876 (25.1)	236 (25.6)	
Household income, No. (%)			
<$20 000	288 (10.1)	27 (3.3)	<.001
$20 000-$40 000	389 (13.9)	63 (7.7)	
$40 000-$60 000	413 (13.8)	91 (11.1)	
≥$60 000	1862 (62.2)	639 (77.9)	
Missing	523	103	
Marital status, No. (%)			
Single	812 (30.5)	107 (13.7)	<.001
Married or living as partners	1771 (61.4)	597 (76.3)	
Widowed, divorced, or separated	245 (8.0)	78 (10.0)	
Missing	647	141	
Education, No. (%)			
Less than high school	54 (2.0)	2 (0.3)	<.001
High school graduate	381 (13.1)	74 (9.4)	
Some college	593 (20.3)	120 (15.3)	
College graduate, postgraduate	1850 (64.5)	590 (75.1)	
Missing	597	137	
Insurance coverage, No. (%)			
Yes	3182 (91.5)	873 (95.1)	<.001
No	272 (8.5)	45 (4.9)	
Missing	21	5	
Age at diagnosis or enrollment, median (IQR), y	7.9 (3.6-13.3)	28.6 (21.7-35.0)	<.001
Diagnosis, No. (%)			
Leukemia	1066 (38.3)	N/A	
CNS tumors	490 (12.5)		
Hodgkin lymphoma	470 (12.0)		
Non-Hodgkin lymphoma	330 (8.4)		
Neuroblastoma	210 (5.4)		
Wilms tumor	327 (8.4)		
Soft tissue sarcoma	249 (6.4)		
Osteosarcoma	205 (5.2)		
Other bone tumors	128 (3.3)		
Anthracycline, No. (%)			
Any	1658 (54.5)	N/A	
None	1604 (45.5)		
Missing	213		
Dose, median (IQR), mg/m²	173.6 (79.3-298.5)		
Alkylating Agent, No. (%)			
Yes	1779 (55.4)	N/A	
No	1477 (44.6)		
Missing	219		
Dose, median (IQR), mg/m²	7412.6 (3727.0-11357.1)		
Bone marrow transplant, No. (%)			
Yes	136 (4.1)	N/A	
No	3137 (95.9)		
Missing	202		
Radiation, No. (%)			
Yes	1839 (53.2)	N/A	
No	1440 (46.8)		
Missing	196		

a All statistics except counts (No.) were accounted for undersampling of acute lymphoblastic leukemia in the expansion (1987-1999) of the Childhood Cancer Survivor Study cohort. CNS = central nervous system; IQR = interquartile range; NA = not applicable.

b Wilcoxon test for continuous variables (testing for medians) or χ^2^ test for categorical variables.

c Race and ethnicity groups categorized following the Surveillance, Epidemiology, and End Results recode categories.

### Neighborhood-level socioeconomic disadvantage and financial hardship

At the time of the financial hardship survey, 1236 (38.7%) survivors resided in an area with high disadvantage (ADI national rank ≥ 50; Table 2), whereas 271 (31.8%) sibling controls resided in an area with high disadvantage (*P *<* *.001). Compared with siblings, there were significantly more survivors living in economically distressed communities (DCI ≥ 80; 11.4% vs 9.0%; *P *=* *.033). There were no significant differences in the associations of sociodemographic factors and area-level deprivation in survivors compared with siblings in linear modeling (ADI quintile) or logistic modeling (ADI national rank ≥ 50).

**Table 2.** Descriptive statistics of area deprivation distribution in survivors and siblings

Distribution measure	Survivors	Siblings	*P* ^a^
	(n = 3475)	(n = 923)	
ADI quintile cutoff points	16, 31, 47, 67		
ADI quintile, No. (%)			
First, least deprivation	604 (18.0)	203 (23.8)	<.001
Second	605 (18.8)	182 (21.4)	
Third	642 (20.5)	172 (20.2)	
Fourth	656 (20.6)	154 (18.1)	
Fifth, greatest deprivation	705 (22.0)	141 (16.5)	
Disadvantage area, No. (%)			
Low disadvantage area, ADI national rank <50	1976 (61.3)	581 (68.2)	
High disadvantage area, ADI national rank ≥50	1236 (38.7)	271 (31.8)	<.001

a Wilcoxon test for continuous variables (testing for medians) or χ^2^ test for categorical variables. ADI = Area Deprivation Index.

**Figure 1.**
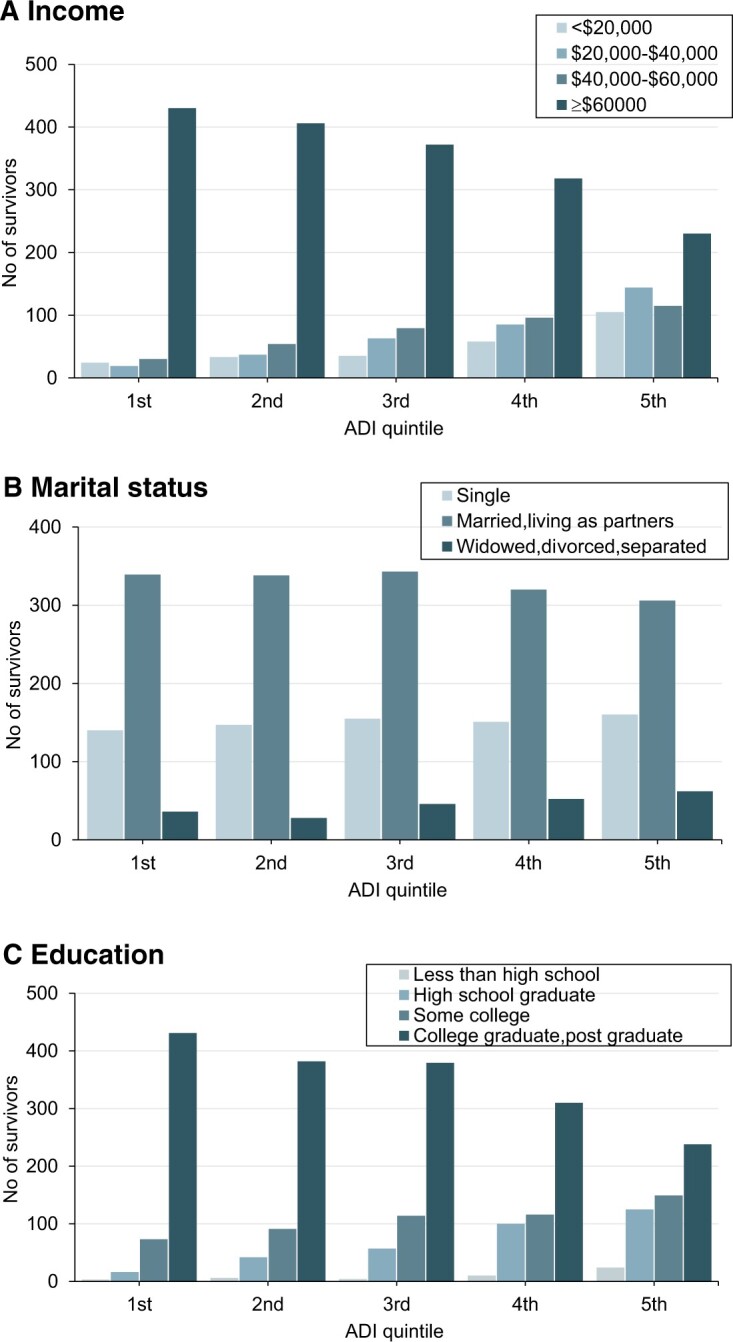
Survivor income **(A)**, marital status **(B)**, and education **(C)** by level of area deprivation (Area Deprivation Index [ADI] quintile). Data presented as number of individuals per category. ADI quintile cutoffs for survivors were as follows: second, 13; third, 27; fourth, 40; fifth, 63.

Bivariate (unadjusted) analyses showed statistically significant associations of individual-level socioeconomic factors (household income, educational attainment, marital status) and level of area deprivation (Figure 1, A-C). Married survivors were more likely to live in low disadvantage areas (63.4% vs 58.5%; *P *=* *.008) than survivors who lived alone (ie, single or divorced or separated). Males had significantly lower behavioral and psychological hardship in survivors and were significantly associated with psychological hardship than siblings (Supplementary Table 2, A and B, available online). As ADI quintile increased, we found statistically significant behavioral and psychological hardship increases, although statistically significant, greater material hardship was observed in the fourth and fifth quintiles only.

**Figure 2.**
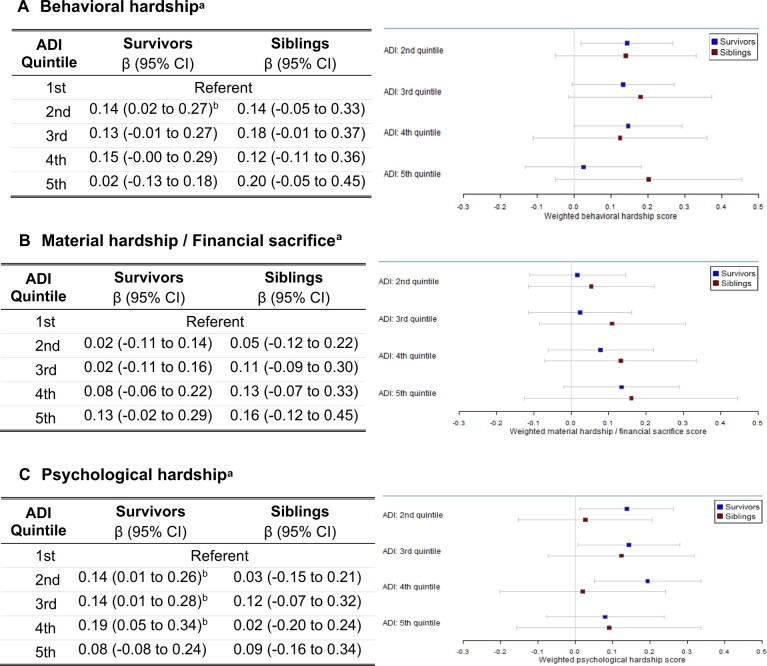
Weighted, adjusted behavioral **(A)**, material and/or financial sacrifice **(B)**, and psychological **(C)** hardship score by level of area deprivation (Area Deprivation Index quintile). Tables and full list of covariates, effect sizes, confidence intervals, and *P* values located in Supplementary Materials (available online). ^a^Analyses accounted for undersampling of acute lymphoblastic leukemia in the expansion (1987-1999) of the Childhood Cancer Survivor Study cohort; analyses were also adjusted for cubic splines (5 knots at age 30, 35, 40, 50, and 55 years) at questionnaire. ^b^*P* < .05. ADI = Area Deprivation Index; CI = confidence interval.

Figure 2 shows the associations between socioeconomic disadvantage and behavioral, material, and psychological hardship for survivors and siblings. For survivors, residing in neighborhoods with increasing area deprivation (ADI quintiles 2-4) was associated with small increases (β ≤ .2) of behavioral (ADI second quintile, *P *=* *.017; Figure 2, A) and psychological hardship (ADI second quintile, *P *=* *.009*;* third quintile, *P *=* *.014*;* see Figure 2, C). When compared with survivors, we observed similar associations of financial hardship domains and levels of area deprivation in siblings except for psychological hardship (Figure 2, C). Siblings in the highest quintile of area deprivation demonstrated moderately lower association with psychological financial hardship (β = -.29) compared with survivors (β = .06), although this was not statistically significant. There was no association between area deprivation and material hardship and financial sacrifice for survivors (Figure 2, B; Supplementary Table 4, A and B, available online). Full survivor and sibling adjusted regression models with coefficients can be found in Supplementary Table 3, A and B, Supplementary Table 4, A and B, and Supplementary Table 5, A and B (available online), respectively.

There were no associations between any DCI quintile level and behavioral, material, and psychological hardship outcomes in survivors and in siblings in adjusted analyses (Supplementary Table 6, available online).

## Discussion

We found long-term survivors of childhood cancer were more likely to reside in areas with greater socioeconomic deprivation than sibling controls and that residence in areas with greater deprivation was associated with financial hardship. Our findings contribute to an emerging area of investigation in the childhood cancer survivor literature (25). We provided evidence that some area levels of socioeconomic disadvantage are modestly associated with financial hardship; individual socioeconomic factors (ie, education, health insurance status) likely influence this association. Survivors who experienced more financial hardship were more likely to live in disadvantaged areas. It is crucial to note that our findings do not attribute financial hardships to residing in disadvantaged areas but rather emphasize the complex portrait of socioeconomic challenges in long-term childhood cancer survivors. Our findings are consistent with evidence among survivors of cancers diagnosed in adulthood, where residing in areas with greater deprivation was associated with financial hardship (26). Others have reported that long-term survivors of childhood cancer are less likely than individuals without a history of cancer to earn a higher income or obtain a higher educational degree and often lack or have inadequate health insurance coverage (15,27,28). Their lower individual SES on average may explain why they reside in higher deprivation areas compared with siblings.

More survivors resided in disadvantaged areas compared with sibling controls, indicating a relationship between cancer in childhood and residence in higher deprivation areas. Our analysis aimed to describe neighborhood-level differences in long-term survivors of childhood cancer. We used the current zip code from a follow-up questionnaire to measure socioeconomic disadvantage (4). We did not aim to determine reasons for residence in disadvantaged areas, which may be unrelated to SES entirely. For example, cancer survivors who experience financial hardship may also lack stable housing—one component of area deprivation (19,29). Approximately 16.6% of adult cancer survivors in the United States within 2 years of our data collection had moderate to elevated levels of housing insecurity (30). Socioeconomic disadvantage may be helpful to describe financial hardship experienced by cancer survivors. However, caution should be exercised when using only neighborhood socioeconomic disadvantage variables, such as area deprivation, to describe disparities, as they do not imply a causal relationship with financial hardship. Individual- and neighborhood-level factors need to be considered together to describe disparities in financial hardship occurring in long-term survivors of childhood cancer.

Our findings suggest a potential association between socioeconomic disadvantage and psychological and behavioral financial hardship. Although access to specialized long-term follow-up clinics is significantly associated with statistically clinically significant improvements in screening for social, psychological, or emotional problems (31,32), there are disparities in access to these programs (33,34). Accreditation standards are increasingly requiring health systems to implement psychological distress screening and management processes, which includes financial topics (35,36). Although we did not study if survivors accessed these programs, evaluating their effectiveness in future work is warranted.

Greater financial hardship among adult survivors of childhood cancer may be explained by the lack of accessible clinical settings where financial assistance and distress screening programs are offered (37). The majority of childhood cancer survivors receive posttreatment care at cancer centers, but later in adulthood, access to financial assistance, distress screening, and specialized survivor programs is often limited to patients undergoing active treatment (37-39). In adult cancer survivors, as many as 63% of National Cancer Institute–designated cancer centers provide medical debt management assistance, and 97% provide assistance with nonmedical costs as of 2023 (40,41). However, services rendered for adult survivors of childhood cancer are few and far between. Programs improving health insurance literacy and financial literacy may mitigate the downstream consequences of financial hardship for adult cancer survivors (42-44).

Although area deprivation focuses on census tract–level socioeconomic adversity, the economic distress measured by the DCI did not show statistically significant associations with any domains of financial hardship. This discrepancy may be due to the use of a broader geographic unit (ie, county) by the DCI to capture neighborhood economic distress. Similar findings were reported using ADI and DCI to examine the association between neighborhood disadvantage with hospital readmissions for adult survivors with colorectal cancer (45). Although association was found between hospital readmission of survivors living in medium to high areas of deprivation, there was no effect of economic distress on financial hardship (45).

This study was subject to limitations. First, although the home address of study participants was available at the same time as financial hardship status was assessed, we were unable to measure the association between survivors’ socioeconomic environment at the time of their initial cancer diagnosis in childhood with financial hardship in adulthood. This study used 2 measures to assess neighborhood socioeconomic characteristics: ADI and DCI. There could be other measures that capture different aspects of neighborhood challenges, and these might provide different insights into how specific aspects of neighborhood challenges are connected to financial hardship. Furthermore, one aspect of the ADI, home value, tends to be overemphasized compared with other factors (46,47). Finally, the cross-sectional nature of the study precluded any formal determination of a causal relationship. Future studies are warranted to collect longitudinal social mobility data to examine a causal relationship of area deprivation with financial hardship in childhood cancer survivors. Despite the limitations, the novelty of this study should be considered. This linkage of CCSS clinical and questionnaire data with neighborhood-level socioeconomic disadvantage data expands our understanding of socioeconomic outcomes in long-term survivors of childhood cancer.

Long-term childhood cancer survivors are likely to face financial hardship that extend into adulthood and are likely to reside in disadvantaged neighborhoods. It is critical to enhance current systems of surveillance by including financial difficulties and socioeconomic disparities as long-term effects of treatments. Improving access to long-term follow-up services could lessen the risk of financial hardships. Grasping the unique financial challenges faced by these survivors, along with the influence of their neighborhood environments, will enable researchers to codevelop targeted and effective social and behavioral interventions. We encourage researchers to foster collaborative strategies that actively shape support and care systems for childhood cancer survivors.

## Supplementary Material

pkae033_Supplementary_Data

## Data Availability

The Childhood Cancer Survivor Study is a US National Cancer Institute funded resource (U24 CA55727) to promote and facilitate research among long-term survivors of cancer diagnosed during childhood and adolescence. CCSS data are publicly available on dbGaP at https://www.ncbi.nlm.nih.gov/gap/ through its accession number phs001327.v2.p1. and on the St Jude Survivorship Portal within the St Jude Cloud at https://survivorship.stjude.cloud/. In addition, utilization of the CCSS data that leverages the expertise of CCSS Statistical and Survivorship research and resources will be considered on a case-by-case basis. For this utilization, a research application of intent followed by an analysis concept proposal must be submitted for evaluation by the CCSS Publications Committee. Users interested in utilizing this resource are encouraged to visit http://ccss.stjude.org. Full analytical datasets associated with CCSS publications since January 2023 are also available on the St Jude Survivorship Portal at https://viz.stjude.cloud/community/cancer-survivorship-community∼4/publications.
