# Supplementary material for: Financial hardship and neighborhood socioeconomic disadvantage in long-term childhood cancer survivors
Source: JNCI Cancer Spectr. 2024 Apr 27;8(3):pkae033. doi: 10.1093/jncics/pkae033 (PMC11126153; doi:10.1093/jncics/pkae033)
Supplement: pkae033_Supplementary_Data [file pkae033_supplementary_data.docx]

**Supplementary Table 1. Financial hardship questionnaire items.**

| **Domain** | **Questionnaire Item** | **Response** |
| --- | --- | --- |
| Behavioral Financial Hardship | Forgone any needed medical | Yes      No |
|  | Forgone yearly visit to primary care doctor |  |
|  | Forgone prescription medicine |  |
|  | Forgone mental health care/counselling |  |
|  | Forgone dental care |  |
|  | Forgone eyeglasses |  |
|  | Forgone Specialist |  |
|  | Forgone follow-up care |  |
|  | Any behavioral hardship |  |
| Material Financial Hardship/  Financial Sacrifices | Reduced spending on vacation or leisure | Yes      No |
|  | Reduced spending for large purchases |  |
|  | Reduced spending on basics |  |
|  | Delayed or reduced spending on home improvement |  |
|  | Used savings set aside for other purposes |  |
|  | Made a change to living situation |  |
|  | Any financial sacrifices |  |
| Psychological Financial Hardship | Worry or stress about having enough money to pay rent or mortgage | Always/Usually/ Sometimes worry      Rarely/Not worry |
|  | Worry or stress about having enough money to buy nutritious meals |  |
|  | Worry or stress about having enough money to pay household utilities, such as water, gas, and electricity |  |
|  | Any psychological hardship |  |

## **Supplementary Table 2A and 2B** (Weighted scores). Multiple adjusted linear regression models on demographics variables only, regressing financial hardship scores on area deprivation for (2A) survivors and (2B) siblings.^a^

|  | **Behavioral Hardship Survivors** | | | | | | | **Material /Financial Hardship Survivors** | | | **Psychological Hardship Survivors** | | |
| --- | --- | --- | --- | --- | --- | --- | --- | --- | --- | --- | --- | --- | --- |
|  | **Effect Size** | | | **β (95% CI)** | ***P* Value** | | | **Effect Size** | **β (95% CI)** | ***P* Value** | **Effect Size** | **β (95% CI)** | ***P* Value** |
| **Area-Level Variables** |  | | | | | | |  | | |  |  |  |
| Area Deprivation (ADI Composite Score) |  | | | | | | |  | | |  |  |  |
| 1^st^ quintile | Reference | | | | | | | Reference | | |  | Reference |  |
| 2^nd^ quintile | 0.12 | | | 0.12 (0.01 - 0.24) | 0.029 | | | 0.04 | 0.04 (-0.06 - 0.15) | 0.428 | 0.15 | 0.15 (0.03 - 0.26) | 0.014 |
| 3^rd^ quintile | 0.21 | | | 0.21 (0.09 - 0.34) | <.001 | | | 0.05 | 0.05 (-0.07 - 0.16) | 0.416 | 0.26 | 0.26 (0.14 - 0.39) | <.001 |
| 4^th^ quintile | 0.27 | | | 0.27 (0.15 - 0.40) | <.001 | | | 0.15 | 0.15 (0.03 - 0.27) | 0.012 | 0.39 | 0.39 (0.26 - 0.51) | <.001 |
| 5^th^ quintile | 0.22 | | | 0.22 (0.10 - 0.35) | <.001 | | | 0.24 | 0.24 (0.12 - 0.35) | <.001 | 0.39 | 0.39 (0.27 - 0.52) | <.001 |
| **Individual Level Variables** |  | | | | | | |  | | |  |  |  |
| Sex |  | | | | | | |  | | |  |  |  |
| Female | Reference | | | | | | | Reference | | |  | Reference |  |
| Male | -0.09 | | -0.09 (-0.17 - -0.01) | | | | 0.030 | -0.07 | -0.07 (-0.15 - 0.01) | 0.073 | -0.14 | -0.14 (-0.22 - -0.06) | <.001 |
| Race/Ethnicity |  | | | | | | |  | | |  |  |  |
| White, non-Hispanic | Reference | | | | | | | Reference | | |  | Reference |  |
| Black, non-Hispanic | -0.18 | -0.18 (-0.40 - 0.03) | | | | 0.099 | | 0.10 | 0.10 (-0.14 - 0.33) | 0.424 | -0.05 | -0.05 (-0.29 - 0.19) | 0.661 |
| Hispanic | -0.03 | -0.03 (-0.19 - 0.14) | | | | 0.772 | | -0.02 | -0.02 (-0.19 - 0.16) | 0.846 | 0.10 | 0.10 (-0.05 - 0.26) | 0.200 |
| Non-Hispanic unknown race | 0.18 | 0.18 (-0.11 - 0.47) | | | | 0.224 | | -0.23 | -0.23 (-0.41 - -0.04) | 0.016 | 0.23 | 0.23 (-0.07 - 0.53) | 0.128 |

^a^ Analyses were accounted for under-sampling of acute lymphoblastic leukemia (ALL) in the expansion cohort (1987-1999) of the CCSS cohort; analyses were also adjusted for cubic splines (five knots at 30, 35, 40, 50 and 55 years) of age at questionnaire.

## **Table 2B - Financial Hardship Scores Siblings**

|  | **Behavioral Hardship Siblings** | | | | | | | **Material /Financial Hardship Siblings** | | | **Psychological Hardship Siblings** | | |
| --- | --- | --- | --- | --- | --- | --- | --- | --- | --- | --- | --- | --- | --- |
|  | **Effect Size** | | | **β (95% CI)** | ***P* Value** | | | **Effect Size** | **β (95% CI)** | ***P* Value** | **Effect Size** | **β (95% CI)** | ***P* Value** |
| **Area-Level Variables** |  | | | | | | |  | | |  |  |  |
| Area Deprivation (ADI Composite Score) |  | | | | | | |  | | |  |  |  |
| 1^st^ quintile | Reference | | | | | | | Reference | | |  | Reference |  |
| 2^nd^ quintile | 0.16 | | | 0.16 (-0.01 - 0.32) | 0.062 | | | 0.03 | 0.03 (-0.13 - 0.19) | 0.705 | 0.10 | 0.10 (-0.06 - 0.26) | 0.205 |
| 3^rd^ quintile | 0.18 | | | 0.18 (0.02 - 0.34) | 0.027 | | | 0.03 | 0.03 (-0.14 - 0.20) | 0.725 | 0.20 | 0.20 (0.03 - 0.37) | 0.018 |
| 4^th^ quintile | 0.19 | | | 0.19 (0.01 - 0.38) | 0.038 | | | 0.10 | 0.10 (-0.08 - 0.28) | 0.273 | 0.24 | 0.24 (0.06 - 0.42) | 0.009 |
| 5^th^ quintile | 0.23 | | | 0.23 (0.05 - 0.41) | 0.013 | | | 0.15 | 0.15 (-0.06 - 0.37) | 0.154 | 0.27 | 0.27 (0.07 - 0.46) | 0.007 |
| **Individual Level Variables** |  | | | | | | |  | | |  |  |  |
| Sex |  | | | | | | |  | | |  |  |  |
| Female | Reference | | | | | | | Reference | | |  | Reference |  |
| Male | -0.02 | | -0.02 (-0.13 - 0.09) | | | | 0.734 | -0.08 | -0.08 (-0.20 - 0.04) | 0.180 | -0.12 | -0.12 (-0.23 - -0.01) | 0.039 |
| Race/Ethnicity |  | | | | | | |  | | |  |  |  |
| White, non-Hispanic | Reference | | | | | | | Reference | | |  | Reference |  |
| Black, non-Hispanic | -0.31 | -0.31 (-0.72 - 0.11) | | | | 0.146 | | -0.10 | -0.10 (-0.60 - 0.41) | 0.709 | -0.17 | -0.17 (-0.44 - 0.10) | 0.227 |
| Hispanic | -0.33 | -0.33 (-0.61 - -0.05) | | | | 0.023 | | 0.06 | 0.06 (-0.24 - 0.37) | 0.676 | -0.25 | -0.25 (-0.51 - 0.01) | 0.060 |
| Non-Hispanic unknown race | -0.18 | -0.18 (-0.47 - 0.12) | | | | 0.236 | | -0.02 | -0.02 (-0.33 - 0.28) | 0.875 | 0.03 | 0.03 (-0.35 - 0.40) | 0.891 |

^a^ Analyses were accounted for under-sampling of acute lymphoblastic leukemia (ALL) in the expansion cohort (1987-1999) of the CCSS cohort; analyses were also adjusted for cubic splines (five knots at 30, 35, 40, 50 and 55 years) of age at questionnaire.

## **Supplementary Table 3A and 3B** (Weighted scores). Multiple unadjusted and adjusted linear regression models, regressing behavioral hardship on area deprivation for (3A) survivors and (3B) siblings.^a^

## **Table 3A** **– Behavioral Hardship Survivors**

|  | **Unadjusted** | | | **Adjusted** | | |
| --- | --- | --- | --- | --- | --- | --- |
|  | **Effect Size** | **β (95% CI)** | ***P* Value** | **Effect Size** | **β (95% CI)** | ***P* Value** |
| **Area-Level Variables** | | | | | | |
| Area Deprivation (ADI Composite Score) | | | | | | |
| 1^st^ quintile | Reference | | | | | |
| 2^nd^ quintile | 0.17 | 0.17 (0.07 - 0.28) | <.001 | 0.14 | 0.14 (0.02 - 0.27) | 0.024 |
| 3^rd^ quintile | 0.25 | 0.25 (0.13 - 0.36) | <.001 | 0.13 | 0.13 (-0.01 - 0.27) | 0.062 |
| 4^th^ quintile | 0.20 | 0.20 (0.06 - 0.34) | 0.006 | 0.15 | 0.15 (-0.00 - 0.29) | 0.051 |
| 5^th^ quintile | 0.27 | 0.27 (0.12 - 0.42) | <.001 | 0.02 | 0.02 (-0.13 - 0.18) | 0.756 |
| **Individual Level Variables** | | | | | | |
| Sex |  | | |  | | |
| Female |  |  |  | Reference | | |
| Male |  |  |  | -0.05 | -0.05 (-0.14 - 0.05) | 0.349 |
| Race/Ethnicity |  |  |  |  | | |
| White, non-Hispanic |  |  |  | Reference | | |
| Black, non-Hispanic |  |  |  | -0.35 | -0.35 (-0.67 - -0.04) | 0.027 |
| Hispanic |  |  |  | -0.16 | -0.16 (-0.35 - 0.04) | 0.118 |
| Non-Hispanic unknown race |  |  |  | 0.15 | 0.15 (-0.11 - 0.41) | 0.249 |
| Income |  |  |  |  | | |
| <$20,000 |  |  |  | Reference | | |
| $20,000-$40,000 |  |  |  | 0.15 | 0.15 (-0.14 - 0.44) | 0.303 |
| $40,000-$60,000 |  |  |  | -0.01 | -0.01 (-0.29 - 0.27) | 0.937 |
| $60,000+ |  |  |  | -0.23 | -0.23 (-0.50 - 0.03) | 0.086 |
| Marital Status |  |  |  |  | | |
| Married/Living as  partners |  |  |  | Reference | | |
| Single |  |  |  | -0.13 | -0.13 (-0.26 - -0.00) | 0.044 |
| Widowed/Divorced/  Separated |  |  |  | 0.01 | 0.01 (-0.22 - 0.24) | 0.950 |
| Education |  |  |  |  | | |
| < High school |  |  |  | Reference | | |
| High School graduate |  |  |  | 0.09 | 0.09 (-0.23 - 0.41) | 0.596 |
| Some college |  |  |  | 0.10 | 0.10 (-0.23 - 0.42) | 0.558 |
| College graduate / Postgraduate |  |  |  | -0.01 | -0.01 (-0.32 - 0.30) | 0.944 |
| Employment Status |  |  |  |  | | |
| Employed/Retired |  |  |  | Reference | | |
| Unemployed/Looking |  |  |  | 0.02 | 0.02 (-0.27 - 0.30) | 0.914 |
| Disabled |  |  |  | -0.07 | -0.07 (-0.23 - 0.09) | 0.361 |
| Insurance Coverage |  |  |  |  | | |
| Yes |  |  |  | -0.59 | -0.59 (-0.85 - -0.32) | <.001 |
| No |  |  |  | Reference | | |
| Diagnosis |  |  |  |  | | |
| Leukemia |  |  |  | Reference | | |
| CNS tumors |  |  |  | -0.00 | -0.00 (-0.19 - 0.18) | 0.965 |
| Hodgkin Lymphoma |  |  |  | -0.01 | -0.01 (-0.17 - 0.14) | 0.879 |
| Non-Hodgkin Lymphoma |  |  |  | -0.17 | -0.17 (-0.35 - 0.01) | 0.059 |
| Neuroblastoma |  |  |  | -0.10 | -0.10 (-0.28 - 0.09) | 0.305 |
| Wilms tumor |  |  |  | -0.14 | -0.14 (-0.32 - 0.04) | 0.135 |
| Soft tissue sarcoma |  |  |  | -0.05 | -0.05 (-0.21 - 0.12) | 0.587 |
| Osteosarcoma |  |  |  | -0.20 | -0.20 (-0.40 - 0.01) | 0.065 |
| Other bone tumors |  |  |  | -0.15 | -0.15 (-0.39 - 0.08) | 0.206 |
| Anthracycline |  |  |  |  | | |
| Any |  |  |  | 0.07 | 0.07 (-0.05 - 0.19) | 0.260 |
| None |  |  |  | Reference | | |
| Alkylating Agents |  |  |  |  | | |
| Any |  |  |  | 0.01 | 0.01 (-0.11 - 0.13) | 0.856 |
| None |  |  |  | Reference | | |
| Radiation |  |  |  |  | | |
| Yes |  |  |  | 0.10 | 0.10 (-0.01 - 0.21) | 0.063 |
| No |  |  |  | Reference | | |

^a^ Analyses were accounted for under-sampling of acute lymphoblastic leukemia (ALL) in the expansion cohort (1987-1999) of the CCSS cohort; analyses were also adjusted for cubic splines (five knots at 30, 35, 40, 50 and 55 years) of age at questionnaire.

## **Table 3B - Behavioral Hardship Siblings**

|  | **Unadjusted** | | | **Adjusted** | | |
| --- | --- | --- | --- | --- | --- | --- |
|  | **Effect Size** | **β (95% CI)** | ***P* Value** | **Effect Size** | **β (95% CI)** | ***P* Value** |
| **Area-Level Variables** | | | | | | |
| Area Deprivation (ADI Composite Score) | | | | | | |
| 1^st^ quintile | Reference | | | | | |
| 2^nd^ quintile | 0.18 | 0.18 (0.05 - 0.32) | 0.008 | 0.14 | 0.14 (-0.05 - 0.33) | 0.153 |
| 3^rd^ quintile | 0.17 | 0.17 (0.01 - 0.34) | 0.041 | 0.18 | 0.18 (-0.01 - 0.37) | 0.069 |
| 4^th^ quintile | 0.24 | 0.24 (0.06 - 0.43) | 0.010 | 0.12 | 0.12 (-0.11 - 0.36) | 0.300 |
| 5^th^ quintile | 0.10 | 0.10 (-0.18 - 0.37) | 0.500 | 0.20 | 0.20 (-0.05 - 0.45) | 0.118 |
| **Individual-Level Variables** | | | | | | |
| Sex |  | | |  | | |
| Female |  |  |  | Reference | | |
| Male |  |  |  | -0.02 | -0.02 (-0.16 - 0.12) | 0.794 |
| Race/Ethnicity |  |  |  |  | | |
| White, non-Hispanic |  |  |  | Reference | | |
| Black, non-Hispanic |  |  |  | -0.22 | -0.22 (-0.74 - 0.30) | 0.409 |
| Hispanic |  |  |  | -0.34 | -0.34 (-0.69 - 0.01) | 0.057 |
| Non-Hispanic unknown race |  |  |  | -0.22 | -0.22 (-0.60 - 0.15) | 0.242 |
| Income |  |  |  |  | | |
| <$20,000 |  |  |  | Reference | | |
| $20,000-$40,000 |  |  |  | 0.08 | 0.08 (-0.54 - 0.69) | 0.806 |
| $40,000-$60,000 |  |  |  | 0.27 | 0.27 (-0.30 - 0.85) | 0.348 |
| $60,000+ |  |  |  | 0.25 | 0.25 (-0.31 - 0.81) | 0.387 |
| Marital status |  |  |  |  | | |
| Married/Living as  partners |  |  |  | Reference | | |
| Single |  |  |  | 0.06 | 0.06 (-0.18 - 0.29) | 0.641 |
| Widowed/Divorced/  Separated |  |  |  | 0.05 | 0.05 (-0.19 - 0.29) | 0.664 |
| Education |  |  |  |  | | |
| < High school |  |  |  | Reference | | |
| High school graduate |  |  |  | 0.65 | 0.65 (-0.19 - 1.49) | 0.128 |
| Some college |  |  |  | 0.58 | 0.58 (-0.25 - 1.40) | 0.170 |
| College graduate/  Postgraduate |  |  |  | 0.69 | 0.69 (-0.13 - 1.51) | 0.098 |
| Employment status |  |  |  |  | | |
| Employed/Retired |  |  |  | Reference | | |
| Unemployed/Looking |  |  |  | 0.28 | 0.28 (-0.15 - 0.72) | 0.203 |
| Disabled |  |  |  | -0.01 | -0.01 (-0.26 - 0.23) | 0.929 |
| Insurance Coverage |  |  |  |  | | |
| Yes |  |  |  | -0.54 | -0.54 (-0.91 - -0.16) | 0.005 |
| No |  |  |  | Reference | | |

^a^ Analyses were accounted for under-sampling of acute lymphoblastic leukemia (ALL) in the expansion cohort (1987-1999) of the CCSS cohort; analyses were also adjusted for cubic splines (five knots at 30, 35, 40, 50 and 55 years) of age at questionnaire.

## **Supplementary Table 4A and 4B** (Weighted Scores). Multiple unadjusted and adjusted linear regression models, regressing material hardship/financial sacrifices on area deprivation for (4A) survivors and (4B) siblings. ^a^

## **Table 4A** - **Material /Financial Hardship Survivors**

|  | **Unadjusted** | | | **Adjusted** | | |
| --- | --- | --- | --- | --- | --- | --- |
|  | **Effect Size** | **β (95% CI)** | ***P* Value** | **Effect Size** | **β (95% CI)** | ***P* Value** |
| **Area-Level Variables** | | | | | | |
| Area Deprivation (ADI Composite Score) | | | | | | |
| 1^st^ quintile | Reference | | | | | |
| 2^nd^ quintile | 0.02 | 0.02 (-0.08 - 0.12) | 0.740 | 0.02 | 0.02 (-0.11 - 0.14) | 0.797 |
| 3^rd^ quintile | 0.07 | 0.07 (-0.04 - 0.18) | 0.198 | 0.02 | 0.02 (-0.11 - 0.16) | 0.734 |
| 4^th^ quintile | 0.19 | 0.19 (0.07 - 0.32) | 0.002 | 0.08 | 0.08 (-0.06 - 0.22) | 0.272 |
| 5^th^ quintile | 0.22 | 0.22 (0.08 - 0.36) | 0.002 | 0.13 | 0.13 (-0.02 - 0.29) | 0.091 |
| **Individual-Level Variables** | | | | | | |
| Sex |  | | |  | | |
| Female |  |  |  | Reference | | |
| Male |  |  |  | -0.10 | -0.10 (-0.19 - -0.00) | 0.040 |
| Race/Ethnicity |  |  |  |  | | |
| White, non-Hispanic |  |  |  | Reference | | |
| Black, non-Hispanic |  |  |  | 0.12 | 0.12 (-0.24 - 0.48) | 0.520 |
| Hispanic |  |  |  | -0.07 | -0.07 (-0.31 - 0.18) | 0.594 |
| Non-Hispanic unknown race |  |  |  | -0.16 | -0.16 (-0.37 - 0.05) | 0.137 |
| Income |  |  |  |  | | |
| <$20,000 |  |  |  | Reference | | |
| $20,000-$40,000 |  |  |  | 0.09 | 0.09 (-0.15 - 0.34) | 0.467 |
| $40,000-$60,000 |  |  |  | 0.12 | 0.12 (-0.12 - 0.36) | 0.337 |
| $60,000+ |  |  |  | -0.17 | -0.17 (-0.39 - 0.05) | 0.129 |
| Marital status |  |  |  |  | | |
| Married/Living as  partners |  |  |  | Reference | | |
| Single |  |  |  | -0.09 | -0.09 (-0.21 - 0.04) | 0.188 |
| Widowed/Divorced/  Separated |  |  |  | 0.06 | 0.06 (-0.14 - 0.26) | 0.546 |
| Education |  |  |  |  | | |
| < High school |  |  |  | Reference | | |
| High school graduate |  |  |  | 0.27 | 0.27 (-0.10 - 0.64) | 0.155 |
| Some college |  |  |  | 0.26 | 0.26 (-0.10 - 0.62) | 0.152 |
| College graduate/  Postgraduate |  |  |  | 0.23 | 0.23 (-0.12 - 0.59) | 0.196 |
| Employment status |  |  |  |  | | |
| Employed/Retired |  |  |  | Reference | | |
| Unemployed/Looking |  |  |  | 0.01 | 0.01 (-0.24 - 0.25) | 0.965 |
| Disabled |  |  |  | 0.13 | 0.13 (-0.02 - 0.27) | 0.080 |
| Insurance coverage |  |  |  |  |  |  |
| Yes |  |  |  | -0.17 | -0.17 (-0.39 - 0.06) | 0.154 |
| No |  |  |  | Reference | | |
| Diagnosis |  |  |  |  |  |  |
| Leukemia |  |  |  | Reference | | |
| CNS tumors |  |  |  | 0.02 | 0.02 (-0.15 - 0.19) | 0.824 |
| Hodgkin Lymphoma |  |  |  | -0.01 | -0.01 (-0.17 - 0.14) | 0.858 |
| Non-Hodgkin Lymphoma |  |  |  | 0.18 | 0.18 (0.01 - 0.34) | 0.033 |
| Neuroblastoma |  |  |  | 0.02 | 0.02 (-0.18 - 0.21) | 0.875 |
| Wilms tumor |  |  |  | 0.07 | 0.07 (-0.11 - 0.26) | 0.428 |
| Soft tissue sarcoma |  |  |  | 0.01 | 0.01 (-0.18 - 0.19) | 0.952 |
| Osteosarcoma |  |  |  | 0.28 | 0.28 (0.07 - 0.50) | 0.010 |
| Other bone tumors |  |  |  | 0.16 | 0.16 (-0.08 - 0.40) | 0.190 |
| Anthracycline |  |  |  |  |  |  |
| Any |  |  |  | -0.01 | -0.01 (-0.13 - 0.11) | 0.905 |
| None |  |  |  | Reference | | |
| Alkylating agents |  |  |  |  |  |  |
| Any |  |  |  | -0.04 | -0.04 (-0.15 - 0.08) | 0.521 |
| None |  |  |  | Reference | | |
| Radiation |  |  |  |  |  |  |
| Yes |  |  |  | 0.11 | 0.11 (0.00 - 0.22) | 0.045 |
| No |  |  |  | Reference | | |

## **Table 4B** **- Material /Financial Hardship Siblings**

|  | **Unadjusted** | | | **Adjusted** | | |
| --- | --- | --- | --- | --- | --- | --- |
|  | **Effect Size** | **β (95% CI)** | ***P* Value** | **Effect Size** | **β (95% CI)** | ***P* Value** |
| **Area-level Variables** | | | | | | |
| Area Deprivation (ADI Composite Score) | | | | | | |
| 1^st^ quintile | Reference | | | | | |
| 2^nd^ quintile | 0.04 | 0.04 (-0.10 - 0.18) | 0.548 | 0.05 | 0.05 (-0.12 - 0.22) | 0.534 |
| 3^rd^ quintile | 0.10 | 0.10 (-0.07 - 0.27) | 0.251 | 0.11 | 0.11 (-0.09 - 0.30) | 0.273 |
| 4^th^ quintile | 0.04 | 0.04 (-0.13 - 0.21) | 0.676 | 0.13 | 0.13 (-0.07 - 0.33) | 0.203 |
| 5^th^ quintile | 0.38 | 0.38 (0.01 - 0.74) | 0.044 | 0.16 | 0.16 (-0.12 - 0.45) | 0.270 |
| **Individual-Level variables** | | | | | | |
| Sex |  | | |  |  |  |
| Female |  |  |  | Reference | | |
| Male |  |  |  | -0.03 | -0.03 (-0.17 - 0.10) | 0.611 |
| Race/Ethnicity |  |  |  |  | | |
| White, non-Hispanic |  |  |  | Reference | | |
| Black, non-Hispanic |  |  |  | 0.15 | 0.15 (-0.51 - 0.80) | 0.661 |
| Hispanic |  |  |  | 0.11 | 0.11 (-0.21 - 0.42) | 0.515 |
| Non-Hispanic unknown race |  |  |  | -0.08 | -0.08 (-0.43 - 0.27) | 0.658 |
| Income |  |  |  |  | | |
| <$20,000 |  |  |  | Reference | | |
| $20,000-$40,000 |  |  |  | 0.23 | 0.23 (-0.31 - 0.78) | 0.403 |
| $40,000-$60,000 |  |  |  | 0.09 | 0.09 (-0.47 - 0.66) | 0.742 |
| $60,000+ |  |  |  | -0.11 | -0.11 (-0.62 - 0.39) | 0.661 |
| Marital status |  |  |  |  | | |
| Married/Living as  partners |  |  |  | Reference | | |
| Single |  |  |  | -0.31 | -0.31 (-0.53 - -0.08) | 0.007 |
| Widowed/Divorced/  Separated |  |  |  | 0.01 | 0.01 (-0.24 - 0.27) | 0.926 |
| Education |  |  |  |  | | |
| < High school |  |  |  | Reference | | |
| High school graduate |  |  |  | 0.41 | 0.41 (-0.06 - 0.87) | 0.087 |
| Some college |  |  |  | 0.36 | 0.36 (-0.05 - 0.77) | 0.089 |
| College graduate/  Postgraduate |  |  |  | 0.56 | 0.56 (0.14 - 0.97) | 0.009 |
| Employment status |  |  |  |  | | |
| Employed/Retired |  |  |  | Reference | | |
| Unemployed/Looking |  |  |  | 0.14 | 0.14 (-0.52 - 0.80) | 0.679 |
| Disabled |  |  |  | 0.18 | 0.18 (-0.08 - 0.43) | 0.172 |
| Insurance coverage |  |  |  |  | | |
| Yes |  |  |  | -0.31 | -0.31 (-0.77 - 0.15) | 0.182 |
| No |  |  |  | Reference | | |

^a^ Analyses were accounted for under-sampling of acute lymphoblastic leukemia (ALL) in the expansion cohort (1987-1999) of the CCSS cohort; analyses were also adjusted for cubic splines (five knots at 30, 35, 40, 50 and 55 years) of age at questionnaire.

## **Supplementary Table 5A and 5B** (Weighted scores). Multiple unadjusted and adjusted linear regression models, regressing psychological hardship on area deprivation for (5A) survivors and (5B) siblings. ^a^

## **Table 5A - Psychological Hardship Survivors**

|  | **Unadjusted** | | | **Adjusted** | | |
| --- | --- | --- | --- | --- | --- | --- |
| *Psychological Hardship* | **Effect Size** | **β (95% CI)** | ***P***  **Value** | **Effect Size** | **β (95% CI)** | ***P* Value** |
| **Area-level Variables** | | | | | | |
| Area Deprivation (ADI Composite Score) | | | | | | |
| 1^st^ quintile | Reference | | | | | |
| 2^nd^ quintile | 0.21 | 0.21 (0.10 - 0.31) | <.001 | 0.14 | 0.14 (0.01 - 0.26) | 0.030 |
| 3^rd^ quintile | 0.34 | 0.34 (0.22 - 0.45) | <.001 | 0.14 | 0.14 (0.01 - 0.28) | 0.038 |
| 4^th^ quintile | 0.35 | 0.35 (0.22 - 0.49) | <.001 | 0.19 | 0.19 (0.05 - 0.34) | 0.008 |
| 5^th^ quintile | 0.47 | 0.47 (0.31 - 0.62) | <.001 | 0.08 | 0.08 (-0.08 - 0.24) | 0.319 |
| **Individual-level Variables** | | | | | | |
| Sex |  | | |  | | |
| Female |  |  |  | Reference | | |
| Male |  |  |  | -0.09 | -0.09 (-0.18 - 0.01) | 0.069 |
| Race/Ethnicity |  |  |  |  | | |
| White, non-Hispanic |  |  |  | Reference | | |
| Black, non-Hispanic |  |  |  | -0.29 | -0.29 (-0.60 - 0.02) | 0.069 |
| Hispanic |  |  |  | 0.06 | 0.06 (-0.12 - 0.24) | 0.524 |
| Non-Hispanic unknown race |  |  |  | 0.20 | 0.20 (-0.10 - 0.49) | 0.186 |
| Income |  |  |  |  | | |
| <$20,000 |  |  |  | Reference | | |
| $20,000-$40,000 |  |  |  | 0.00 | 0.00 (-0.26 - 0.26) | 0.996 |
| $40,000-$60,000 |  |  |  | -0.32 | -0.32 (-0.58 - -0.06) | 0.015 |
| $60,000+ |  |  |  | -0.81 | -0.81 (-1.05 - -0.57) | <.001 |
| Marital status |  |  |  |  | | |
| Married/Living as  partners |  |  |  | Reference | | |
| Single |  |  |  | -0.12 | -0.12 (-0.24 - -0.00) | 0.046 |
| Widowed/Divorced/  Separated |  |  |  | 0.00 | 0.00 (-0.20 - 0.20) | 0.999 |
| Education |  |  |  |  | | |
| < High school |  |  |  | Reference | | |
| High school graduate |  |  |  | 0.04 | 0.04 (-0.35 - 0.42) | 0.851 |
| Some college |  |  |  | 0.07 | 0.07 (-0.30 - 0.45) | 0.705 |
| College graduate/  Postgraduate |  |  |  | -0.12 | -0.12 (-0.49 - 0.26) | 0.542 |
| Employment status |  |  |  |  | | |
| Employed/Retired |  |  |  | Reference | | |
| Unemployed/Looking |  |  |  | 0.03 | 0.03 (-0.22 - 0.27) | 0.831 |
| Disabled |  |  |  | -0.18 | -0.18 (-0.32 - -0.03) | 0.019 |
| Insurance coverage |  |  |  |  |  |  |
| Yes |  |  |  | -0.27 | -0.27 (-0.51 - -0.04) | 0.025 |
| No |  |  |  | Reference | | |
| Diagnosis |  |  |  |  | | |
| Leukemia |  |  |  | Reference | | |
| CNS tumors |  |  |  | 0.04 | 0.04 (-0.14 - 0.21) | 0.684 |
| Hodgkin Lymphoma |  |  |  | -0.01 | -0.01 (-0.16 - 0.14) | 0.897 |
| Non-Hodgkin Lymphoma |  |  |  | -0.09 | -0.09 (-0.26 - 0.07) | 0.269 |
| Neuroblastoma |  |  |  | -0.04 | -0.04 (-0.23 - 0.15) | 0.662 |
| Wilms tumor |  |  |  | -0.12 | -0.12 (-0.28 - 0.05) | 0.172 |
| Soft tissue sarcoma |  |  |  | 0.02 | 0.02 (-0.14 - 0.19) | 0.770 |
| Osteosarcoma |  |  |  | -0.11 | -0.11 (-0.31 - 0.10) | 0.316 |
| Other bone tumors |  |  |  | -0.04 | -0.04 (-0.27 - 0.19) | 0.712 |
| Anthracycline |  |  |  |  | | |
| Any |  |  |  | 0.06 | 0.06 (-0.05 - 0.17) | 0.284 |
| None |  |  |  | Reference | | |
| Alkylating agents |  |  |  |  | | |
| Any |  |  |  | 0.00 | 0.00 (-0.11 - 0.11) | 0.947 |
| None |  |  |  | Reference | | |
| Radiation |  |  |  |  | | |
| Yes |  |  |  | 0.04 | 0.04 (-0.07 - 0.14) | 0.502 |
| No |  |  |  | Reference | | |

## **Table 5B** **- Psychological Hardship Siblings**

|  | **Unadjusted** | | | **Adjusted** | | |
| --- | --- | --- | --- | --- | --- | --- |
|  | **Effect Size** | **β (95% CI)** | ***P***  **Value** | **Effect Size** | **β (95% CI)** | ***P* Value** |
| **Area-level Variables** | | | | | | |
| Area Deprivation (ADI Composite Score) | | | | | | |
| 1^st^ quintile | Reference | | | | | |
| 2^nd^ quintile | 0.16 | 0.16 (0.02 - 0.29) | 0.024 | 0.03 | 0.03 (-0.15 - 0.21) | 0.773 |
| 3^rd^ quintile | 0.19 | 0.19 (0.03 - 0.36) | 0.024 | 0.12 | 0.12 (-0.07 - 0.32) | 0.219 |
| 4^th^ quintile | 0.34 | 0.34 (0.16 - 0.53) | <.001 | 0.02 | 0.02 (-0.20 - 0.24) | 0.859 |
| 5^th^ quintile | 0.04 | 0.04 (-0.23 - 0.30) | 0.789 | 0.09 | 0.09 (-0.16 - 0.34) | 0.476 |
| **Individual-level Variables** | | | | | | |
| Sex |  | | |  | | |
| Female |  |  |  | Reference | | |
| Male |  |  |  | -0.11 | -0.11 (-0.25 - 0.03) | 0.114 |
| Race/Ethnicity |  |  |  |  | | |
| White, non-Hispanic |  |  |  | Reference | | |
| Black, non-Hispanic |  |  |  | -0.20 | -0.20 (-0.60 - 0.20) | 0.323 |
| Hispanic |  |  |  | -0.26 | -0.26 (-0.55 - 0.03) | 0.075 |
| Non-Hispanic unknown race |  |  |  | -0.01 | -0.01 (-0.44 - 0.42) | 0.961 |
| Income |  |  |  |  | | |
| <$20,000 |  |  |  | Reference | | |
| $20,000-$40,000 |  |  |  | -0.26 | -0.26 (-0.88 - 0.35) | 0.400 |
| $40,000-$60,000 |  |  |  | -0.08 | -0.08 (-0.66 - 0.50) | 0.786 |
| $60,000+ |  |  |  | -0.45 | -0.45 (-1.02 - 0.11) | 0.114 |
| Marital status |  |  |  |  | | |
| Married/Living as  partners |  |  |  | Reference | | |
| Single |  |  |  | 0.07 | 0.07 (-0.16 - 0.30) | 0.566 |
| Widowed/Divorced/  Separated |  |  |  | 0.02 | 0.02 (-0.23 - 0.27) | 0.872 |
| Education |  |  |  |  | | |
| < High school |  |  |  | Reference | | |
| High school graduate |  |  |  | 0.35 | 0.35 (-0.09 - 0.79) | 0.115 |
| Some college |  |  |  | 0.33 | 0.33 (-0.08 - 0.75) | 0.115 |
| College graduate/  Postgraduate |  |  |  | 0.36 | 0.36 (-0.03 - 0.75) | 0.073 |
| Employment status |  |  |  |  | | |
| Employed/Retired |  |  |  | Reference | | |
| Unemployed/Looking |  |  |  | 0.24 | 0.24 (-0.24 - 0.72) | 0.320 |
| Disabled |  |  |  | -0.00 | -0.00 (-0.25 - 0.24) | 0.977 |
| Insurance coverage |  |  |  |  | | |
| Yes |  |  |  | -0.18 | -0.18 (-0.55 - 0.19) | 0.347 |
| No |  |  |  | Reference | | |

^a^ Analyses were accounted for under-sampling of acute lymphoblastic leukemia (ALL) in the expansion cohort (1987-1999) of the CCSS cohort; analyses were also adjusted for cubic splines (five knots at 30, 35, 40, 50 and 55 years) of age at questionnaire.

**Supplementary Table 6** Multiple unadjusted linear regression models, regressing behavioral, material, and psychological hardship (weighted scores) on county-level Distressed Communities Index (DCI) quintiles for (A) survivors and (B) sibling controls.^a^

|  | **Survivors** | | | **Sibling Controls** | | |
| --- | --- | --- | --- | --- | --- | --- |
|  | Effect Size | β (95% CI) | *P* Value | Effect Size | β (95% CI) | *P* Value |
| **Distressed Communities Index** | | | |  | | |
| Behavioral Hardship | | | |  | | |
| 1^st^ quintile | Reference | | | Reference | | |
| 2^nd^ quintile | 0.14 | 0.14 (-0.34 - 0.61) | 0.571 | -0.03 | -0.03 (-0.74 - 0.67) | 0.923 |
| 3^rd^ quintile | 0.38 | 0.38 (-0.13 - 0.88) | 0.145 | -0.44 | -0.44 (-1.26 - 0.38) | 0.291 |
| 4^th^ quintile | -0.10 | -0.10 (-0.64 - 0.43) | 0.707 | 0.01 | 0.01 (-0.93 - 0.96) | 0.978 |
| 5^th^ quintile | 0.39 | 0.39 (-0.23 - 1.01) | 0.216 | -0.19 | -0.19 (-1.18 - 0.80) | 0.708 |
| Material Hardship | | | |  | | |
| 1^st^ quintile | Reference | | | Reference | | |
| 2^nd^ quintile | -0.13 | -0.13 (-0.53 - 0.28) | 0.545 | 0.05 | 0.05 (-0.57 - 0.67) | 0.884 |
| 3^rd^ quintile | **-0.54** | **-0.54 (-0.96 - -0.12)** | **0.011** | -0.35 | -0.35 (-1.05 - 0.34) | 0.319 |
| 4^th^ quintile | -0.09 | -0.09 (-0.53 - 0.34) | 0.675 | -0.59 | -0.59 (-1.42 - 0.24) | 0.164 |
| 5^th^ quintile | -0.28 | -0.28 (-0.80 - 0.24) | 0.291 | -0.76 | -0.76 (-1.62 - 0.10) | 0.083 |
| Psychological Hardship | | | |  | | |
| 1^st^ quintile | Reference | | | Reference | | |
| 2^nd^ quintile | 0.37 | 0.37 (-0.08 - 0.82) | 0.110 | -0.01 | -0.01 (-0.67 - 0.65) | 0.975 |
| 3^rd^ quintile | **0.53** | **0.53 (0.05 - 1.01)** | **0.030** | -0.17 | -0.17 (-0.97 - 0.62) | 0.672 |
| 4^th^ quintile | 0.09 | 0.09 (-0.45 - 0.62) | 0.755 | 0.74 | 0.74 (-0.15 - 1.63) | 0.103 |
| 5^th^ quintile | **0.63** | **0.63 (0.03 - 1.23)** | **0.040** | -0.05 | -0.05 (-0.99 - 0.89) | 0.920 |

^a^ Analyses were accounted for under-sampling of acute lymphoblastic leukemia (ALL) in the expansion cohort (1987-1999) of the CCSS cohort; analyses were also adjusted for cubic splines (five knots at 30, 35, 40, 50 and 55 years) of age at questionnaire.

Bolded values represent *P* < .05.
